# Expression of NALPs in adipose and the fibrotic progression of non-alcoholic fatty liver disease in obese subjects

**DOI:** 10.1186/s12876-014-0208-8

**Published:** 2014-12-16

**Authors:** Rohini Mehta, Arpan Neupane, Lei Wang, Zachary Goodman, Ancha Baranova, Zobair M Younossi

**Affiliations:** Betty and Guy Beatty Obesity and Liver Program, Inova Health System, Falls Church, VA USA; Center for the Study of Chronic Metabolic Diseases, School of Systems Biology, College of Science, George Mason University, Fairfax, VA USA; Center for Liver Diseases and Department of Medicine, Inova Fairfax Hospital, Falls Church, VA USA

**Keywords:** Obesity, NAFLD, Inflammasomes, Cytokines, Fibrosis

## Abstract

**Background:**

Visceral obesity is often accompanied by non-alcoholic fatty liver disease (NAFLD). Activation of NACHT, LRR and PYD domains-containing proteins (NALPs) may contribute to the release of pro-inflammatory cytokines by adipose and the obesity-associated progression of NAFLD to non-alcoholic steatohepatitis (NASH).

**Methods:**

We analyzed visceral adipose expression of various NALPs and its downstream effectors caspase-1, ASC (Apoptosis-associated speck-like protein containing a CARD), IL-18 (Interleukin-18) and IL-1β (Interleukin- 1Beta) in obese subjects (BMI ≥ 35) with biopsy proven NAFLD.

**Results:**

In adipose samples collected from NASH and pericellular fibrosis patients cohorts, expression levels of NALPs and IL-1β were lower than that in non-NASH patients. In portal fibrosis, the levels of mRNA encoding anti-inflammatory NALP6 were upregulated. The expression levels of all NALPs were significantly co-correlated. Circulating IL-18 levels were associated with increased liver injury markers AST and ALT and portal fibrosis.

**Conclusion:**

Our observations point at a possible shift in inflammation and fibrotic response from adipose tissue to liver and a possible negative feedback regulation of tissue inflammation that may instigate NAFLD severity.

**Electronic supplementary material:**

The online version of this article (doi:10.1186/s12876-014-0208-8) contains supplementary material, which is available to authorized users.

## Background

The prevalence of obesity in United States has remained unabated over the past 10 years with ~35% prevalence among adults [1]. A pro-inflammatory state (also referred to as low-grade chronic inflammation/meta-inflammation/sterile inflammation) associated with visceral obesity, has been also shown to be strongly correlated with the development of non-alcoholic fatty liver disease (NAFLD) [2]. This non-pathogen associated pro-inflammatory state can be stimulated and propagated by a variety of damage-associated molecular patterns (DAMPs) that originate from damaged tissue and/or tissue under stress [3-5]. Once released, DAMPs bind to pattern-recognition receptors (PRRs) to elicit an immune response by promoting the release of pro-inflammatory mediators and by recruiting the immune cells to the tissue.

Among the five main classes of vertebrate PRRs, the largest receptor family is comprised by nucleotide oligomerization and binding domain (NOD)-like receptors (NLRs) with a conserved NOD motif [6]. NLRs are believed to be the most evolutionarily ancient family of PRRs [7]. NACHT, LRR and PYD domains-containing proteins (NLRPs/NALPs) belong to the NOD-like receptor (NLR) family that differs from other NLRs by their N-terminal PYD domains [8,9].

Recently NALPs have attracted attention as PRRs that link recognition of DAMPs with the regulation of inflammatory response [7,10]. Human genome encodes 14 NALPs, some of which are required for inflammasome formation [9]. The inflammasomes are large, signal-induced multiprotein complexes responsible for the proteolytic cleavage and activation of procaspases-1. In turn, activated caspase-1 promotes maturation of the pro-inflammatory cytokines interleukin-18 (IL-18), interleukin-1β (IL-1β) and interleukin-33 (IL-33) [10,11,12]. The NALP1 inflammasome is composed of NALP1, apoptosis-associated speck-like protein containing a carboxy-terminal CARD (ASC), caspase-1, and caspase-5, whereas the NALP2/3 inflammasome contains NALP2 or NALP3, CARDINAL, ASC, and caspase-1 [13].

The downstream targets of inflammasomes include known regulators of inflammation and immunity- IL-1β and IL-18. IL-1β triggers the production of interleukin-6 (IL-6) and tumor necrosis factor-alpha (TNF-α), two cytokines that elicits immune cell migration and infiltration into tissue [14], and promote the generation and maintenance of interferon-gamma (IFN-γ) and interleukin-17 (IL-17) producing T cells [15]. Interleukin-18 (IL-18) incites immune cell recruitment and activation [11] and influences natural killer (NK) cell and T cell effector responses [16,17].

A large number of recent studies have shown adipose tissue to be the major source of inflammatory molecules in obesity [18-21]. The expression patterns of NALPs have not been previously explored in visceral adipose. It is reasonable to speculate that NALPs may be activated in adipose tissue of obese individuals and that these NALPs may contribute to the release of pro-inflammatory IL-1β and IL-18 from adipocytes, the development of the systemic inflammation and the obesity-associated progression of non-alcoholic fatty liver disease (NAFLD) to non-alcoholic steatohepatitis (NASH).

With this in mind, we profiled visceral adipose expression of 14 NALPs as well as caspase-1, ASC, IL-18 and IL-1β in 45 obese subjects (BMI ≥ 35) with biopsy proven NAFLD. Expression levels of NALPs were correlated to the serum indicators of system-wide inflammation IL-1β and IL-18 and to various parameters describing the underlying liver pathology.

## Methods

### Sample collection

This study has been approved by Internal Review Board of Inova Fairfax Hospital. After written informed consent, omental adipose tissue and serum samples from 45 obese patients (BMI = 47 ± 11) undergoing bariatric surgery were collected. The samples were immediately flash frozen in liquid nitrogen and added to the repository of specimens stored at −80°C. The samples were de-identified in compliance with HIPAA regulations. For each patient, a liver biopsy was also performed and subjected to histopathological evaluation. Clinical data were available for all the samples in the repository and included pre-surgery fasting glucose, serum aminotransferases (alanine aminotransferase (ALT) and aspartate aminotransferase (AST)) and lipid panel. None of the included subjects reported to have excessively consumed alcohol (>10 grams/day in women and >20 grams/day in men) in the past 5 years. Other chronic liver diseases were excluded by negative serology for hepatitis B and C, no history of toxic exposure and no other cause of chronic liver disease.

### Pathological assessment

After staining with hematoxylin-eosin or Masson trichrome, the slides were reviewed by single hepatopathologist (Z.G.) that followed a predetermined histologic grading system [22] that included quantitative evaluation of the fibrosis and various inflammatory features.

### Definitions and scoring

Steatosis was histologically defined as the presence of >5% hepatic fat with or without lobular inflammation and with/without portal inflammation and graded on a scale of 0–3 as follows: 0 = none, 1 ≤ 5%, 2 = 6-33%, 3 = 34-66%, 4 = > 66%. Immune cell infiltrates (lymphoplasmacytic cells, Kupffer cell hypertrophy and neutrophil presence) were scored on a score of 0–3 as follows: 0 = none, 1 = mild or few, 2 = moderate, 3 = marked or many. Lobular inflammation was defined by sum of scores of the presence of lymphoplasmacytic cells, Kupffer cell hypertrophy and neutrophil infiltration. Portal inflammation, hepatocellular ballooning, pericellular/perisinusoidal fibrosis, and portal fibrosis were graded on a scale of 0 to 3: (0) none, (1) mild or few, (2) moderate, or (3) marked or many. Advanced fibrosis was defined by a sum of score of portal and pericellular fibrosis as being greater or equal to 3. Bridging fibrosis was scored as (0) none, (1) few bridges, or (2) many bridges. Cirrhosis was scored as (0) absent, (1) incomplete, or (2) established. NASH was histologically defined as steatosis, lobular inflammation, and ballooning degeneration with or without Mallory Denk bodies, and with or without pericellular fibrosis. Patients who had hepatic steatosis (with or without lobular inflammation) or NASH were considered to have NAFLD [22].

### RNA extraction and reverse transcription

Total RNA was extracted from visceral adipose tissue (100 mg) using Aurum Total RNA Fatty and Fibrous Tissue Kit (Bio-Rad, USA) and eluted in 30 uL of RNase free water (Fisher Scientific, USA). The quantity and purity of the extracted RNA was determined by absorbance at 260 nm (A260) and 280 nm (A280) measured by the GeneQuant1300 spectrophotometer (GE Healthcare, USA). A260/A280 ratio between 1.8 and 2.1 was considered as an indicator of high quality RNA. The integrity of total RNA was verified by 1% agarose gel electrophoresis with ethidium bromide (10 μg/ml). The results were documented using Molecular Imager (Bio-Rad, USA). All total RNA samples demonstrated a 2:1 intensity ratio of sharp, clear 28S and 18S rRNA bands.

To prevent deterioration of RNA during storage, cDNA synthesis was carried out on the same day as total RNA extraction using RT^2^ HT First Strand Kit (Qiagen). According to manufacturer’s protocol, 1 ug of total RNA was subjected to a genomic DNA elimination step prior to reverse transcription. Reverse transcription was carried out in the presence of random hexamers and oligo-dT and resultant cDNA preps were diluted to a final volume of 111 uL.

### Quantitative RT-PCR

Validated primers for specific amplification of NALP1-NALP14, Caspase-1, ASC, IL-1B and IL-18 mRNA were as described in relevant publications (see Additional file 1: Table S1). The specificity of each primer was verified using NCBI BLAST [23] and the correct size of PCR product was confirmed by gel electrophoresis in 2% agarose. For the purpose of normalization, visceral adipose validated housekeeping gene ACTB was used as the reference [24].

Quantitative real-time PCR was performed in a 96 well format in the Bio-Rad CFX96 Real Time System (Bio-Rad, USA). The real-time PCR mixture consisted of 1 μL cDNA corresponding to 1 ug of total RNA, 250 nM primers and 1× Sso Fast Evagreen Supermix (BioRad, USA) in a final volume of 10 μL. Each plate included no template control to detect reagent contamination. Each run also included wells with TATA box binding protein (TBP) primer pair (Invitrogen, USA) and universal cDNA (Qiagen, USA) as both an interplate control and a positive control. The thermal profile of the RT-PCR procedure repeated for 50 cycles was: 1) 95°C for 30s; 2) 5 s denaturation at 95°C, 5 s annealing at 60°C (amplification data collected at the end of each amplification step); 3) dissociation curve consisting of 10 s incubation at 95°C, 5 s incubation at 65°C, a ramp up to 95°C (Bio-rad CFX96 Real Time System, USA). Melting curves were used to validate product specificity. All samples were amplified in triplicates from the same total RNA preparation and the mean value was used for further analysis. Ct values of target genes greater than 37 were considered to be a negative call and assigned a value = 37 for the purpose of normalization. Ct values of control wells (no-template control, positive control) were examined for each plate.

### ELISA

Serum IL-18 levels were measured by Human IL-18 ELISA kit (R&D systems, USA) according to manufacturer’s instructions. The limits of detection of the assay were at 12.5 pg/mL. Active serum IL-1β levels were measured using Quantikine HS Human IL-1β immunoassay (R&D systems, USA) according to manufacturer’s instructions. The sensitivity ranges from 0.023 pg/mL to 0.14 pg/mL. The limits of detection in this assay was at 0.125 pg/mL.

### Data analysis

Among samples that differed in their histologically determined severity scores, group comparisons (Table 1) were performed using non-parametric Mann–Whitney *U* test. Spearman’s correlation analysis was carried out. In all cases, the p- values of < 0.05 were considered to be significant.

**Table 1:** **Demographic and clinical characteristics of patient cohorts profiled for expression of NALPs and other inflammasome components and targets**

**Demographic or clinical parameter (N = 45)**	**Average ± SD or percentage**
Clinical & Demographic Data	
BMI	47.4 ± 10.8
Age	42.7 ± 11.9
Gender (Females)	62.2%
Triglyceride (mg/dL)	158.3 ± 96.7
AST (U/L)	27.7 ± 18.8
ALT (U/L)	36.7 ± 29.3
Glucose (mg/dL)	106.4 ± 32.2
Diabetes (Presence)	57.7%
Liver Histological Data	
Non-NASH NAFLD	55.5%
NASH (Presence)	44.4%
Ballooning Degeneration (Presence)	28.8%
Mallory-Denk Bodies (Presence)	11.1%
Portal Inflammation (Presence)	66.6%
Advanced Lobular Inflammation (Score ≥ 3)	28.8%
Pericellular Fibrosis (Presence)	44.4%
Portal Fibrosis (Presence)	75.5%
Advanced Fibrosis (Score ≥ 3)	31.1%
Focal Necrosis (Presence)	31.1%

Steatosis is histologically defined by the presence of ≥ 5% hepatic fat with/without lobular inflammation and with/without portal inflammation. The degree of lobular inflammation is defined by the sum of the scores for the presence of lymphoplasmacytic cells, Kupffer cell hypertrophy and neutrophil infiltration. NASH is histologically defined by the presence of steatosis along with lobular inflammation, with/without ballooning degeneration and/or Mallory-Denk bodies and/or pericellular fibrosis. Lymphoplasmacytic cells, Kupffer cell hypertrophy and neutrophil infiltration was each scored on a scale of 0–3: 0 = none, 1 = mild or few, 2 = moderate, 3 = marked or many. Portal inflammation, hepatocellular ballooning, pericellular/perisinusoidal fibrosis, and portal fibrosis were graded on a scale of 0 to 3: (0) none, (1) mild or few, (2) moderate, or (3) marked or many. Advanced fibrosis was defined by a sum of score of portal and pericellular fibrosis as being greater or equal to 3. Bridging fibrosis was scored as (0) none, (1) few bridges, or (2) many bridges. Cirrhosis was scored as (0) absent, (1) incomplete, or (2) established [22].

## Results

### NALP4 and IL-1B encoding mRNAs are downregulated in visceral adipose of patients with pericellular fibrosis

Pericellular fibrosis scores were positively correlated with both the scores for portal fibrosis (r = 0.5321; p = 0.0003) and the degree of hepatic steatosis (r = 0.3393; p = 0.03001). In visceral adipose of patients with pericellular fibrosis, the levels of NALP4-encoding mRNA were significantly lower than that in the cohort without the pericellular fibrosis (0.18 ± 0.20 vs 1.12 ± 2.53; p < 0.019) (Table 2). The levels of mRNA for an inflammatory cytokine IL-1B were also significantly lower in the group with pericellular fibrosis (6.17 ± 12.05 vs 14.96 ± 18.21; p < 0.003) (Table 2). Accordingly, adipose specific expression levels of NALP4 and IL1B mRNAs negatively correlated with pericellular fibrosis (r = −0.33; p < 0.037 and r = −0.47; p = 0.002, respectively), while circulating levels of IL-18 cytokine were positively correlated with the scores for this histopathological feature (r = 0.3365; p < 0.04).

**Table 2:** **Significantly altered targets in analyzed cohorts**

	**Pericellular fibrosis presence (N = 20)**	**Pericellular fibrosis absence (N = 25)**	**P value**
AST (U/L)	34.75 ± 25.06	22.62 ± 9.19	0.023
Males	50.0%	19.0%	0.036
Females	35.7%	25%	NS
Advanced fibrosis presence (Score ≥ 3)	60%	9.5%	0.0006
NALP4 mRNA	0.18 ± 0.20	1.12 ± 2.53	0.019
IL1B mRNA	6.17 ± 12.05	14.96 ± 18.21	0.003
	**Portal fibrosis presence (N = 34)**	**Portal fibrosis absence (N = 11)**	**P value**
Advanced fibrosis presence (Score ≥ 3)	45.2%	0	0.008
NASH	58.1%	20%	0.03
NALP6 mRNA	0.86 ± 0.93	0.38 ± 0.38	0.028
IL-18 (pg/mL)	390.95 pg/mL ± 176.84	246.08 pg/mL ± 103.10	0.022
	**NASH (N = 20)**	**Non-NASH NAFLD (N = 25)**	**P value**
Gender (Females)	50%	81%	< 0.036
Portal Fibrosis (presence)	1.35 ± 0.67	0.71 ± 0.64	< 0.005
Pericellular Fibrosis (presence)	1.45 ± 0.60	0.05 ± 0.22	< 0.001
Advanced fibrosis (Score ≥ 3)	60%	9.5%	< 0.0006
Degree of lobular inflammation	2.55 ± 1.61	1.52 ± 0.98	< 0.02
AST (U/L)	35.70 ± 24.79	21.71 ± 8.52	< 0.004
NALP 4 mRNA	0.16 ± 0.19	1.14 ± 2.53	< 0.002
NALP 5 mRNA	0.14 ± 0.12	1.24 ± 2.58	< 0.02
NALP 7 mRNA	0.14 ± 0.12	1.09 ± 2.54	< 0.03
NALP 8 mRNA	0.14 ± 0.12	1.09 ± 2.54	< 0.03
NALP 9 mRNA	0.14 ± 0.12	1.25 ± 2.70	< 0.03
NALP 10 mRNA	0.14 ± 0.12	1.09 ± 2.54	< 0.03
NALP 11 mRNA	0.14 ± 0.12	1.09 ± 2.54	< 0.02
NALP 13 mRNA	0.14 ± 0.12	1.09 ± 2.54	< 0.03
IL-1B mRNA	8.71 ± 15.45	12.66 ± 16.68	< 0.02

### NALP6 encoding mRNA and serum levels of IL-18 are upregulated in portal fibrosis

Expression levels of NALP6 mRNA were significantly higher in visceral adipose of patients with portal fibrosis as compared to that of the patients with no evidence of portal fibrosis (0.86 ± 0.93 vs 0.38 ± 0.38; p = 0.028) (Table 2). Interestingly, circulating levels of IL-18, an inflammatory cytokine that functions downstream of inflammasome, were significantly higher in patients with portal fibrosis (390.95 pg/mL ± 176.84 pg/mL vs 246.08 pg/mL ± 103.10 pg/mL; p = 0.022) (Table 2). Accordingly, circulating levels of IL-18 were also positively correlated with the scores for portal fibrosis (r = 0.4226; p < 0.01).

### Adipose inflammasome signature associated with inflammatory features and NASH

Among the genes assessed for expression, NALP 4, 5, 7, 8, 9, 10, 11, 13 and IL-1B were significantly downregulated in adipose tissue samples collected from NASH cohort (Table 2).

CASP-1 mRNA expression levels in adipose were positively correlated with both the hepatic lobular inflammation scores (r = 0.3713; p = 0.016) and the histopathologically determined Kupffer cell hypertrophy (r = 0.41; p = 0.006). Additionally, expression levels of NALP3 mRNA in adipose were positively correlated with serum levels of liver enzyme AST (r = 0.32; p = 0.03).

### Inflammasome components are co-regulated in adipose

The pattern of the correlation between expression levels for mRNAs encoding inflammasome components and its downstream targets (NALP1-14, ASC, CASP-1, IL-1B and IL-18 mRNA) shows that these genes are co-regulated in adipose (Table 3). In our study, expression levels of NALP1 mRNA positively correlated with that of NALP4 (r = 0.3984; p = 0.01), NALP11 (r = 0.3294; p = 0.04) and NALP14 (r = 0.4134; p = 0.008). Expression levels of NALP2 mRNAs were positively correlated with that of all the other non-inflammasome forming NALP members (NALP4 - NALP14) as well as with levels of IL-18 mRNA (r = 0.5241, p < 0.001). The expression levels of NALP3 mRNA were correlated with that of NALP5 (r = 0.3188, p = 0.04), NALP12 (r = 0.3662, p = 0.02), NALP14 (r = 0.3484, p = 0.027) as well as ASC (r = 0.78, p < 0.001) and CASP-1 (r = 0.389, p = 0.012). The levels of NALP4 mRNA were co-correlated with levels of NALP5, NALP7-14 and IL-18 mRNA (Table 3), while levels of NALP5 mRNA were co-correlated with NALP7-14 and IL-18 mRNAs (Table 3). NALP6 mRNA levels were co-correlated with that of NALP12 (r = 0.3227, p = 0.048). The levels mRNAs for NALP7, NALP8, NALP9 and NALP10 were co-correlated with NALP11, NALP12, NALP14 and IL-18 mRNA (Table 3). The levels of NALP11 mRNA were co-correlated with NALP12/13/14 and IL-18 mRNA, the levels of NALP12 co-correlated with NALP13, NALP14, ASC and IL-18 mRNA, and the levels of NALP13 co-correlated with NALP14 and IL-18 mRNA. The levels of NALP14 were co-correlated with that of IL-18 mRNA, while the levels of ASC were co-correlated with that of CASP-1 and IL-1B mRNAs.

**Table 3:** **Correlation among mRNA expression levels of inflammasome components**

**Rho (p-value)**	**NALP1**	**NALP2**	**NALP3**	**NALP4**	**NALP5**	**NALP6**	**NALP7**	**NALP8**	**NALP9**	**NALP10**	**NALP11**	**NALP12**	**NALP13**	**NALP14**	**IL18**	**ASC**	**CASP1**	**IL1B**
NALP1	-	-	-	0.3984 (0.01)	-	-	-	-	-	-	0.3294 (0.04)	-	-	0.4134 (0.008)	-	-	-	-
NALP2	-	-	-	0.5336 (<0.001)	0.5068 (<0.001)	0.394 (0.01)	0.5274 (<0.001)	0.5274 (<0.001)	0.5274 (<0.001)	0.5274 (<0.001)	0.5096 (<0.001)	0.5231 (<0.001)	0.5274 (<0.001)	0.4799 (0.001)	-	-	-	-
NALP3	-	-	-	-	0.3188 (0.044)	-	-	-	-	-	-	0.3662 (0.02)	-	0.3484 (0.027)	-	0.7826 (<0.001)	0.389 (0.012)	-
NALP4	-	-	-	-	0.8199 (<0.0001)	-	0.8375 (<0.0001)	0.8375 (<0.0001)	0.8375 (<0.0001)	0.8375 (<0.0001)	0.8786 (<0.0001)	0.6051 (<0.0001)	0.8375 (<0.0001)	0.8317 (<0.0001)	0.3937 (0.01)	-	-	-
NALP5	-	-	-	-	-	-	0.9856 (<0.0001)	0.9856 (<0.0001)	0.9856 (<0.0001)	0.9856 (<0.0001)	0.9786 (<0.0001)	0.7439 (<0.0001)	0.9856 (<0.0001)	0.8829 (<0.0001)	0.564 (<0.0001)	-	-	-
NALP6	-	-	-	-	-	-	-	-	-	-	-	0.3227	-	-	-	-	-	-
NALP7	-	-	-	-	-	-	-	-	-	-	0.9931 (<0.0001)	0.7672 (<0.0001)	-	0.9023 (<0.0001)	0.5581 (<0.0001)	-	-	-
NALP8	-	-	-	-	-	-	-	-	-	-	0.9931 (<0.0001)	0.7672 (<0.0001)	-	0.9023 (<0.0001)	0.5581 (<0.001)	-	-	-
NALP9	-	-	-	-	-	-	-	-	-	-	0.9931 (<0.0001)	0.7672 (<0.0001)	-	0.9023 (<0.0001)	0.5581 (<0.001)	-	-	-
NALP10	-	-	-	-	-	-	-	-	-	-	0.9931 (<0.0001)	0.7672 (<0.0001)	-	0.9023 (<0.0001)	0.5581 (<0.001)	-	-	-
NALP11	-	-	-	-	-	-	-	-	-	-	-	0.758 <0.0001)	0.9931 (<0.0001)	0.9212 (<0.0001)	0.5478 (<0.001)	-	-	-
NALP12	-	-	-	-	-	-		-	-	-	-	-	0.7672 (<0.0001)	0.8437 (<0.0001)	0.514 (<0.001)	0.3657 (0.02)	-	-
NALP13	-	-	-	-	-	-	-	-	-	-	-	-	-	0.9023 (<0.0001)	0.5581 (<0.001)	-	-	-
NALP14	-	-	-	-	-	-	-	-	-	-	-	-	-	-	0.5751 (0.0001)	-	-	-
IL18	-	-	-	-	-	-	-	-	-	-	-	-	-	-	-	-	-	-
ASC	-	-	-	-	-	-	-	-	-	-	-	-	-	-	-	-	-	0.3931 (0.01)
CASP	-	-	-	-	-	-	-	-	-	-	-	-	-	-	-	-	-	-
IL1B	-	-	-	-	-	-	-	-	-	-	-	-	-	-	-	-	-	-

## Discussion

Systemic inflammation is a common finding in both visceral obesity and chronic liver disease [25,26]. Inflammasome activation has been recently recognized to play an increasingly important role in the development of liver disease [27-29]. However, the tissue source and mechanisms of inflammasome mediated liver damage in obesity associated NAFLD remain disputed [29,30]. Activation of inflammasomes involves two separate steps, the priming of tissue to inflammatory response that results in transcriptional activation of NALPs, IL18 and IL1B genes, and the activation of the inflammasome complex that culminates in the secretion of active pro-inflammatory IL-1β and IL-18 cytokines. To better understand the role of adipose specific inflammasome components in NAFLD, we assessed the adipose tissue mRNA for inflammasome components and serum levels of its downstream targets in 45 morbidly obese patients (BMI ≥ 35) with various liver conditions of NAFLD spectrum.

Previous studies have shown that the NALP3 inflammasome and NALP3-dependent caspase-1 activation are central to the inflammation and the development of the liver fibrosis [31]. In our study, expression levels of NALP3 mRNA in adipose were positively correlated with serum levels of liver enzyme AST(r = 0.32; p = 0.03) and the levels of mRNA encoding caspase-1 were positively correlated with Kupffer cell hypertrophy and the hepatic lobular inflammation scores. However, to our surprise, the levels of mRNA encoding for NALP 4, 5, 7, 8, 9, 10, 11, 13 and IL-1B were significantly downregulated in adipose tissue samples collected from NASH cohort as compared to patients without NASH (Table 2). Moreover, the expression patterns of mRNA for many NALPs, especially for non-inflammasome forming NALPs, were co-correlated to each other (Table 3).

So far, only NALPs 1–3 have been shown to form active inflammasome, while the functions of other NALPs remain obscure [9]. Some NALP proteins, for example, NALP12 and NALP6, have been previously shown to suppress inflammation through downregulation of NF-κB signaling subsequent to TLR activation [3]. Another revolving theme in the study of non-classic NALPs, for example, NALP5, NALP7, NALP8 and NALP9 is their involvement in oocyte maturation, implantation and other fertility-related processes [32,33]. It is tempting to speculate that the decrease in adipocytic expression of these NALPs may contribute to decreased fertility in patients with NASH and metabolic syndrome, possibly through known association of NASH with polycystic ovary syndrome (PCOS) [34,35]. Non-inflammasome related NALPs may also play as yet unknown roles in pathogenesis of obesity-associated disorders through modulation of adipocytic secretion or other pathways.

Interestingly, mRNAs encoding for NALP4 and its substrate IL1B were both downregulated in the cohort with hepatic pericellular fibrosis, while recently published study of Boaru et al. showed time and concentration-dependent increase in expression of NALP4 in cultured hepatic stellate cells upon lipopolysacharride (LPS) stimulation [29]. This would suggest a role for NALP4 in promoting fibrotic response by activated stellate cells. The contradiction between our data and observation of Boaru et al. [29] may be attributed to either yet unknown tissue specific differences in the function of NALP4, or to a negative feedback signal operating between adipose tissue and liver which may be, in fact, time dependent. It is possible that the inflammation and the fibrotic processes may be initiated in adipose tissue as described Reggio et al., [36], but later be shifted to distant organs such as liver. This may be accompanied with negative feedback signaling that originates in the liver and attempts to restore homeostasis.

**Figure 1:**
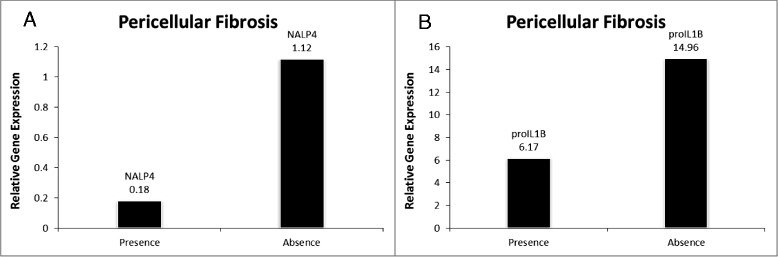
**Significantly altered gene expression in presence of pericellular fibrosis vs absence of pericellular fibrosis. A.)** NALP4 gene expression; **B.)** IL1B gene expression.

**Figure 2:**
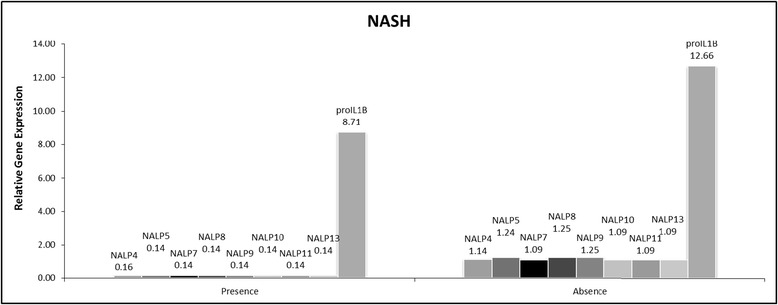
**Significantly altered gene expression in presence of NASH vs non-NASH NAFLD.**

The observed downregulation of adipose-specific production of IL1B mRNA in patients with pericellular fibrosis (Table 2 and Figure 1) as well as NASH (Table 2 and Figure 2) also supports this hypothesis. The downregulation at expression level is also supported by the lack of detectable IL1B protein levels in circulation (Assay Range: 0.125 - 8 pg/mL). Unlike the production of most inflammatory cytokines, the production of biologically active IL-1β is dependent on transcription, translation, maturation and secretion mechanisms, all of which are tightly regulated in tissue-specific manner. This may be attributed to tissue specific roles of IL1B. Animal studies showed that hepatic IL-1β protein and mRNA levels to be increased in various diet-induced NASH models in mice [28], while adipose specific IL1B deficiency in mice increase susceptibility to obesity [28,37]. In another recent study, the authors demonstrate in animal models that IL-1β supports ectopic fat accumulation in hepatocytes and adipose-tissue macrophages, contributing to impaired fat-liver crosstalk in nutritional obesity [38]. While the translation of animal studies to humans is difficult, the discrepancy in observed expression levels of IL1B in obesity associated NASH and pericellular fibrosis maybe attributed to the extremely high BMI of the cohort being examined (BMI ≥ 35). This along with the observed negative correlation of IL1B mRNA levels with BMI (r = −0.317; p = 0.04) indicates an ongoing negative feedback loop between adipose specific IL1B expression and an accumulation of VAT.

**Figure 3:**
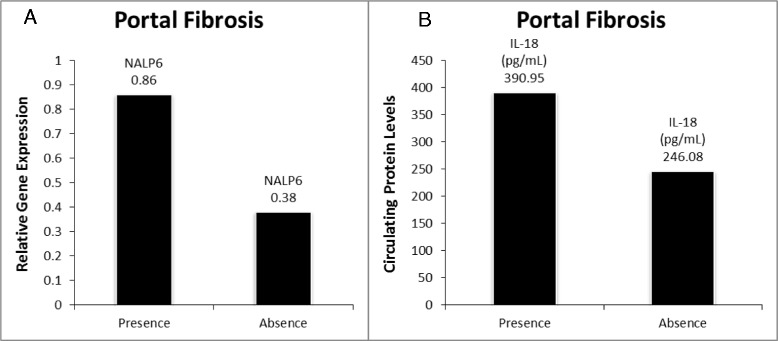
**Significantly altered targets in presence of portal fibrosis vs absence of portal fibrosis. A.)** NALP6 gene expression; **B.)** Circulating IL18 levels.

Further support to the hypothesis centering on ongoing negative feedback loop operating between adipose tissue and liver in morbidly obese individuals with severe NAFLD, is provided by result of the comparison in another cohort. In cohort of subjects with portal fibrosis compared to those without portal fibrosis, adipose specific NALP6 mRNA (Table 2 and Figure 3) and circulating IL18 (Table 2 and Figure 3) protein were upregulated with hepatic portal fibrosis, while adipose specific IL18 mRNA was not significantly different in the same cohort. This is interesting, since NALP6 has been shown to have an anti-inflammatory role by downregulating NF-κB signaling subsequent to TLR activation [3]. Conspicuously, whilst circulating IL-18 protein was upregulated in portal fibrosis (Table 2 and Figure 3), this was not accompanied with an upregulation of adipose specific IL18 mRNA. Additionally, circulating IL-18 was found to be positively correlated with BMI (r = 0.41; p = 0.012). This is in agreement with previous studies [39-43]. Notably, circulating IL-18 levels are also associated with increased liver injury markers [44]: AST (r = 0.33; p = 0.04) and ALT (r = 0.41; p = 0.01) levels respectively as seen in previous reports [43]. Since IL-18 is more widely expressed, this may indicate additional sources of circulating IL-18 protein such as from the gut [28] or the liver [45], playing a role in the inflammation and hepatic injury progression. Thus, IL-18 might contribute to the development of liver disease, albeit the origin of IL-18 may not be solely from adipose.

For the first time, we profiled gene expression profile of all the 14 members of NALPs in visceral adipose tissue. As can be seen from correlation analysis (Table 3), the expression of these genes is significantly correlated with each other. While most of the studies on obesity and insulin resistance have focused on NALP3 mediated inflammasome, our study showed that there is a need for exploring roles of other members of this family of proteins in systemic inflammation and chronic liver conditions.

Among the limitations of this study is that the gene expression analysis has been carried out in whole adipose tissue instead of studying isolated tissue components, i.e. adipocytes and stromal vascular cells. This study was limited to two markers of systemic inflammation, IL1B and IL18 that were selected as known targets released into circulation upon activation. The profiling of greater variety of inflammatory molecules may yield better mechanistic resolution of inflammatory responses in adipose. Another avenue to explore is the parallel study of NALP component expression in liver and other peripheral tissues, and the study of NALP components at protein level.

## Conclusion

The results of our study indicate that non-inflammasome related NALPs may play as yet unknown roles in pathogenesis of obesity-associated disorders through modulation of adipocytic secretion or other pathways. Our observations also point at a possible shift in inflammation and fibrotic response from adipose tissue to liver in patients with severe obesity. While this hypothesis needs further experimental verification, it takes us a step closer in understanding the underlying reason for some obese individuals being metabolically healthy as compared to metabolically unhealthy obese. Additional study of matched liver and adipose tissues from same individuals will help delineate the cross talk mechanisms between inflammation and fibrosis in obesity and NAFLD.

## Additional file

Additional file 1: Table S1. A list of validated primers.

## Abbreviations

NAFLD: Non-alcoholic fatty liver disease
NALPs: NACHT, LRR and PYD domains-containing proteins
NASH: Non-alcoholic steatohepatitis
ASC: Apoptosis-associated speck-like protein containing a CARD
IL-18: Interleukin-18
IL-1β: Interleukin- 1Beta
BMI: Body mass index
AST: Aspartate aminotransferase
ALT: Alanine aminotransferase
DAMPs: Damage-associated molecular patterns
PRRs: Pattern-recognition receptors
NLRs: Nucleotide oligomerization and binding domain NOD-like receptors
IL-33: Interleukin-33
IL-6: Interleukin-6
TNF-α: Tumor necrosis factor-alpha
IFN-γ: Interferon-gamma
IL-17: Interleukin-17
PCOS: Polycystic ovary syndrome

## References

[1] Flegal KM, Caroll MD (2012). PRevalence of obesity and trends in the distribution of body mass index among us adults, 1999–2010. JAMA.

[2] Kubes P, Mehal WZ (2012). Sterile inflammation in the liver. Gastroenterology.

[3] Kono H, Rock KL (2008). How dying cells alert the immune system to danger. Nat Rev Immunol.

[4] Rock KL, Latz E, Ontiveros F, Kono H (2010). The sterile inflammatory response. Annu Rev Immunol.

[5] Maslanik T, Mahaffey L, Tannura K, Beninson L, Greenwood BN, Fleshner M (2013). The inflammasome and danger associated molecular patterns (DAMPs) are implicated in cytokine and chemokine responses following stressor exposure. Brain Behav Immun.

[6] Wilmanski JM, Petnicki-Ocwieja T, Kobayashi KS (2008). NLR proteins: integral members of innate immunity and mediators of inflammatory diseases. J Leukoc Biol.

[7] Lupfer C, Kanneganti T-D (2013). The expanding role of NLRs in antiviral immunity. Immunol Rev.

[8] Martinon F, Gaide O, Pétrilli V, Mayor A, Tschopp J (2007). NALP Inflammasomes: a central role in innate immunity. Semin Immunopathol.

[9] Tschopp J, Martinon F, Burns K (2003). NALPs: a novel protein family involved in inflammation. Nat Rev Mol Cell Biol.

[10] Bryan NB, Dorfleutner A, Rojanasakul Y, Stehlik C (2009). Activation of inflammasomes requires intracellular redistribution of the apoptotic speck-like protein containing a caspase recruitment domain. J Immunol.

[11] Dinarello CA (1998). Interleukin-1, interleukin-1 receptors and interleukin-1 receptor antagonist. Int Rev Immunol.

[12] Martinon F, Burns K, Tschopp J (2002). The inflammasome: a molecular platform triggering activation of inflammatory caspases and processing of proIL-beta. Mol Cell.

[13] Martinon F, Tschopp J (2006). Inflammatory caspases and inflammasomes: master switches of inflammation. Cell Death Differ.

[14] Sims JE, Smith DE (2010). The IL-1 family: regulators of immunity. Nat Rev Immunol.

[15] Ben-Sasson SZ, Hogg A, Hu-Li J, Wingfield P, Chen X, Crank M, Caucheteux S, Ratner-Hurevich M, Berzofsky JA, Nir-Paz R, Paul WE (2013). IL-1 enhances expansion, effector function, tissue localization, and memory response of antigen-specific CD8 T cells. J Exp Med.

[16] Dinarello CA (1994). The interleukin-1 family: 10 years of discovery. FASEB J.

[17] Nakanishi K, Yoshimoto T, Tsutsui H, Okamura H (2001). Interleukin-18 is a unique cytokine that stimulates both Th1 and Th2 responses depending on its cytokine milieu. Cytokine Growth Factor Rev.

[18] Baranova A, Collantes R, Gowder SJ, Elariny H, Schlauch K, Younoszai A, King S, Randhawa M, Pusulury S, Alsheddi T, Ong JP, Martin LM, Chandhoke V, Younossi ZM (2005). Obesity-related differential gene expression in the visceral adipose tissue. Obes Surg.

[19] Lago F, Dieguez C, Gómez-Reino J, Gualillo O (2007). Adipokines as emerging mediators of immune response and inflammation. Nat Clin Pract Rheumatol.

[20] Powell K (2007). Obesity: the two faces of fat. Nature.

[21] Gerner RR, Wieser V, Moschen AR, Tilg H (2013). Metabolic inflammation: role of cytokines in the crosstalk between adipose tissue and liver1. Can J Physiol Pharmacol.

[22] Younossi ZM, Stepanova M, Rafiq N, Makhlouf H, Younoszai Z, Agrawal R, Goodman Z (2011). Pathologic criteria for nonalcoholic steatohepatitis: interprotocol agreement and ability to predict liver-related mortality. Hepatology.

[23] Ye J, Coulouris G, Zaretskaya I, Cutcutache I, Rozen S, Madden TL (2012). Primer-BLAST: a tool to design target-specific primers for polymerase chain reaction. BMC Bioinformatics.

[24] Mehta R, Birerdinc A, Hossain N, Afendy A, Chandhoke V, Younossi Z, Baranova A (2010). Validation of endogenous reference genes for qRT-PCR analysis of human visceral adipose samples. BMC Mol Biol.

[25] Berg AH, Scherer PE (2005). Adipose tissue, inflammation, and cardiovascular disease. Circ Res.

[26] Fontana L, Eagon JC, Trujillo ME, Scherer PE, Klein S (2007). Visceral fat adipokine secretion is associated with systemic inflammation in obese humans. Diabetes.

[27] Benetti E, Chiazza F, Patel NSA, Collino M (2013). The NLRP3 inflammasome as a novel player of the intercellular crosstalk in metabolic disorders. Mediat Inflamm.

[28] Szabo G, Csak T (2012). Inflammasomes in liver diseases. J Hepatol.

[29] Boaru SG, Borkham-Kamphorst E, Tihaa L, Haas U, Weiskirchen R (2012). Expression analysis of inflammasomes in experimental models of inflammatory and fibrotic liver disease. J Inflamm.

[30] Henao-Mejia J, Elinav E, Jin C, Hao L, Mehal WZ, Strowig T, Thaiss CA, Kau AL, Eisenbarth SC, Jurczak MJ, Camporez J-P, Shulman GI, Gordon JI, Hoffman HM, Flavell RA (2012). Inflammasome-mediated dysbiosis regulates progression of NAFLD and obesity. Nature.

[31] Wree A, Eguchi A, McGeough MD, Pena CA, Johnson CD, Canbay A, Hoffman HM, Feldstein AE (2014). NLRP3 inflammasome activation results in hepatocyte pyroptosis, liver inflammation and fibrosis. Hepatology.

[32] Ponsuksili S, Brunner RM, Goldammer T, Kühn C, Walz C, Chomdej S, Tesfaye D, Schellander K, Wimmers K, Schwerin M (2006). Bovine NALP5, NALP8, and NALP9 genes: assignment to a QTL region and the expression in adult tissues, oocytes, and preimplantation embryos. Biol Reprod.

[33] Zhang P, Dixon M, Zucchelli M, Hambiliki F, Levkov L, Hovatta O, Kere J (2008). Expression analysis of the NLRP gene family suggests a role in human preimplantation development. PLoS ONE.

[34] Baranova A, Tran TP, Afendy A, Wang L, Shamsaddini A, Mehta R, Chandhoke V, Birerdinc A, Younossi ZM (2013). Molecular signature of adipose tissue in patients with both non-alcoholic fatty liver disease (NAFLD) and polycystic ovarian syndrome (PCOS). J Transl Med.

[35] Baranova A, Tran TP, Birerdinc A, Younossi ZM (2011). Systematic review: association of polycystic ovary syndrome with metabolic syndrome and non-alcoholic fatty liver disease. Aliment Pharmacol Ther.

[36] Reggio S, Pellegrinelli V, Clément K, Tordjman J (2013). Fibrosis as a cause or a consequence of white adipose tissue inflammation in obesity. Curr Obes Rep.

[37] García MC, Wernstedt I, Berndtsson A, Enge M, Bell M, Hultgren O, Horn M, Ahrén B, Enerback S, Ohlsson C, Wallenius V, Jansson J-O (2006). Mature-onset obesity in interleukin-1 receptor I knockout mice. Diabetes.

[38] Kotas ME, Jurczak MJ, Annicelli C, Gillum MP, Cline GW, Shulman GI, Medzhitov R (2013). Role of caspase-1 in regulation of triglyceride metabolism. PNAS.

[39] Nov O, Shapiro H, Ovadia H, Tarnovscki T, Dvir I, Shemesh E, Kovsan J, Shelef I, Carmi Y, Voronov E, Apte RN, Lewis E, Haim Y, Konrad D, Bashan N, Rudich A (2013). Interleukin-1β regulates fat-liver crosstalk in obesity by auto-paracrine modulation of adipose tissue inflammation and expandability. PLoS ONE.

[40] Bruun JM, Stallknecht B, Helge JW, Richelsen B (2007). Interleukin-18 in plasma and adipose tissue: effects of obesity, insulin resistance, and weight loss. Eur J Endocrinol.

[41] Esposito K, Pontillo A, Ciotola M, Di Palo C, Grella E, Nicoletti G, Giugliano D (2002). Weight loss reduces interleukin-18 levels in obese women. J Clin Endocrinol Metab.

[42] Membrez M, Ammon-Zufferey C, Philippe D, Aprikian O, Monnard I, Macé K, Darimont C (2009). Interleukin-18 protein level is upregulated in adipose tissue of obese mice. Obesity.

[43] Wang H-N, Wang Y-R, Liu G-Q, Liu Z, Wu P-X, Wei X-L, Hong T-P (2008). Inhibition of hepatic interleukin-18 production by rosiglitazone in a rat model of nonalcoholic fatty liver disease. World J Gastroenterol.

[44] Paschos P, Paletas K (2009). Non alcoholic fatty liver disease and metabolic syndrome. Hippokratia.

[45] Finotto S, Siebler J, Hausding M, Schipp M, Wirtz S, Klein S, Protschka M, Doganci A, Lehr HA, Trautwein C, Khosravi-Far R, Strand D, Lohse A, Galle PR, Blessing M, Neurath MF, Khosravi-Fahr R (2004). Severe hepatic injury in interleukin 18 (IL-18) transgenic mice: a key role for IL-18 in regulating hepatocyte apoptosis in vivo. Gut.

