# Supplementary material for: Expression of NALPs in adipose and the fibrotic progression of non-alcoholic fatty liver disease in obese subjects
Source: BMC Gastroenterol. 2014 Dec 16;14:208. doi: 10.1186/s12876-014-0208-8 (PMC4279907; doi:10.1186/s12876-014-0208-8)
Supplement: Additional file 1: Table S1. — A list of validated primers. [file 12876_2014_208_MOESM1_ESM.doc]

**Table S**1: A list of validated primers.

| **Target Gene** | **Sequence** | **Reference** |
| --- | --- | --- |
| *NALP1* | F-5’AAGACCAGCTGTTCTCGGAGTT  R-5’AGGCATGAGATCTCCTGGTTTC | (Zhang et al., 2008) |
| *NALP2* | F-5’TGAGGAAACCACTGTGCAACTT  R-5’AACTGAACGGAGGGATGGAA | (Zhang et al., 2008) |
| *NALP3* | F-5’GAAGAAAGATTACCGTAAGAAGTACAGAAA  R-5’CGTTTGTTGAGGCTCACACTCT | (Zhang et al., 2008) |
| *NALP4* | F-5’AACTACCCAGCAGGCAACGT  R-5’AATCAATGGGTGAGAGGTGACAA | (Zhang et al., 2008) |
| *NALP5* | F-5’CGAGGTCATGAGAGAACCATCTT  R-5’CACGCGGCGGTGAGA | (Zhang et al., 2008) |
| *NALP6* | F-5’GACCCTCAGTCTGGCCTCTGT  TCCGGCTTTGCTCTCTTCAC | (Zhang et al., 2008) |
| *NALP7* | F-5’CTTCTGTGCGGATTCTTTGTGA  R-5’TTTTTAATCTCCACTTTCTGCAGATG | (Zhang et al., 2008) |
| *NALP8* | F-5’AGGCACCCTCAGTGCAAACT  R-5’CCCGTCAAAACACCGATTAAG | (Zhang et al., 2008) |
| *NALP9* | F-5’CGCATGTGTGTGGAGAATATCTTT  R-5’CCCGCCAGTAGACGAGCTT | (Zhang et al., 2008) |
| *NALP10* | F-5’CAAGGGCTTGAAGGTCATGAA  R-5’CGCACATGCTCTCGGTATACTT | (Zhang et al., 2008) |
| *NALP11* | F-5’CGCACACTCAAGTTGTCCTATGTC  R-5’ACGAGCCAAAGCCTTGAGTAAG | (Zhang et al., 2008) |
| *NALP12* | F-5’CCAGAAACTGTGGCTGGATAGC  R-5’GCGTTGTTGGTCAGGTAAAGG | (Zhang et al., 2008) |
| *NALP13* | F-5’CTCTGAAACCACATCGTGCATT  R-5’GCAAGCAGTTGTCAGATTGCAT | (Zhang et al., 2008) |
| *NALP14* | F-5’TCAGAGGCTCGGGTTGGA  R-5’TGCAGATAAGAGCAGAGGAGAGATC | (Zhang et al., 2008) |
| *ASC* | F-5’GCCGAGGAGCTCAAGAAGTT  R-5’CAGGCTGGTGTGAAACTGAA | (Shigeoka et al., 2010) |
| *CASP-1* | F-5’TCCAATAATGGACAAGTCAAGCC  R-5’GCTGTACCCCAGATTTTGTAGCA | (Motani et al., 2011) |
| *IL18* | F-5’CCAAGGAAATCGGCCTCTAT  R-5’CCCCCAATTCATCCTCTTTT | (Chen et al., 2011) |
| *IL-1B* | F-5’AGCTGATGGCCCTAAACAGA  R-5’TCTTTCAACACGCAGGACAG | (Gandapu et al., 2011) |
